# SCN2A-linked myelination deficits and synaptic plasticity alterations drive auditory processing disorders in ASD

**DOI:** 10.21203/rs.3.rs-4925935/v1

**Published:** 2024-08-28

**Authors:** Jun Hee Kim, Han-Gyu Bae, Wan-Chen Wu, Kaila Nip, Elizabeth Gould

**Affiliations:** University of Michigan; University of Michigan; UT Health San Antonio; UT Health San Antonio

## Abstract

Autism spectrum disorder (ASD) is a neurodevelopmental disorder characterized by complex sensory processing deficits. A key unresolved question is how alterations in neural connectivity and communication translate into the behavioral manifestations seen in ASD. Here, we investigate how oligodendrocyte dysfunction alters myelin plasticity and neuronal activity, leading to auditory processing disorder associated with ASD. We focus on the *SCN2A* gene, an ASD-risk factor, to understand its role in myelination and neural processing within the auditory nervous system. Through transcriptional profiling, we identified alterations in the expression of myelin-associated genes in *Scn2a* conditional knockout mice, highlighting the cellular consequences engendered by *Scn2a* deletion in oligodendrocytes. The results reveal a nuanced interplay between oligodendrocytes and axons, where *Scn2a* deletion causes alterations in the intricate process of myelination. This disruption instigates changes in axonal properties, presynaptic excitability, and synaptic plasticity at the single cell level. Furthermore, oligodendrocyte-specific *Scn2a* deletion compromises the integrity of neural circuitry within auditory pathways, leading to auditory hypersensitivity. Our findings reveal a novel pathway linking myelin deficits to synaptic activity and sensory abnormalities in ASD.

## Introduction

A prevailing hypothesis suggests that ASD emanates from alterations in brain connectivity^1–3^. Myelination is critical for brain connectivity and temporal processing in the developing brain by coordinating effective axonal conduction and neurotransmission^4–6^. Recent studies using neuroimaging and genetic analysis have shown atypical white matter development and abnormal myelination in individuals with ASD and animal models ^7, 8^. However, the specific impact of myelin alternations on neural connectivity, communication, and ultimately behavioral manifestations is not fully delineated. Notably, white matter integrity was altered in specific brain regions of humans with ASD, which are associated with sensory processing^9, 10^. Auditory processing abnormalities including auditory hypersensitivity are well-documented in individual with ASD^9^. Auditory hypersensitivity, as an excessive or abnormal response to auditory stimuli, may arise from alterations in sensory gain, neuronal activity, and excitation/inhibition (E/I) balance in the auditory circuitry ^11–13^. These alterations could be potentially linked to abnormalities in myelination.

ASD has been associated with altered gene expression related to oligodendrocyte (OL) maturation and myelination^8^. OLs, the myelin producing glia, play a more complex role in neural development. Beyond facilitating myelination, immature OLs monitor neuronal activities and influence their functional development by modulating axonal segments^14, 15^. Moreover, OLs engage in dynamic interaction with neurons, contributing to activity-dependent myelination, which in turn supports neural circuit plasticity^16–18^. Thus, OL dysfunction can disrupt neuron-glia interactions and alter ion channel expression along the axon, ultimately affecting neural plasticity^5, 19^. However, the impact of OL dysfunction on neuronal activity and synaptic plasticity at the single-cell level as well as how these alterations contribute to auditory processing deficits in ASD remains largely unexplored. Additionally, the genetic and cellular interactions of OLs underpinning the neurodevelopmental feature of ASD such as auditory processing deficits require further studied.

One gene of particular interest is *Scn2a*, which encodes the alpha subunit of the voltage-gated Na^+^ channel 1.2 (Na_v_1.2), is highly linked to neurodevelopmental disorders including ASD^20–22^. Loss-of-function mutations in *Scn2a* impair dendritic excitability, leading to synaptic dysfunction and behavioral deficits^22–24^. Expression of *Scn2a* in non-neuronal cells, specifically in oligodendroglia, has been well documented^25–27^. Notably, a transcriptional profile of OL lineage cells from mouse brain showed the highest levels of *Scn2a* expression are in newly formed OLs, an immature OL population, and a subpopulation of *Scn2a*-expressing OLs exhibits membrane excitability during early postnatal development^27^. However, the cell autonomous role of *Scn2a* in myelination and neuron-glia interaction related to ASD remains to be elucidated. In this study, we investigated how *Scn2a* deletion specifically in OLs impacts the interplay between myelination and synaptic activity, ultimately leading to auditory processing disorders in ASD.

## Results

### Scn2a cKO mice exhibit auditory hypersensitivity without changes in peripheral function.

**Figure 1:**
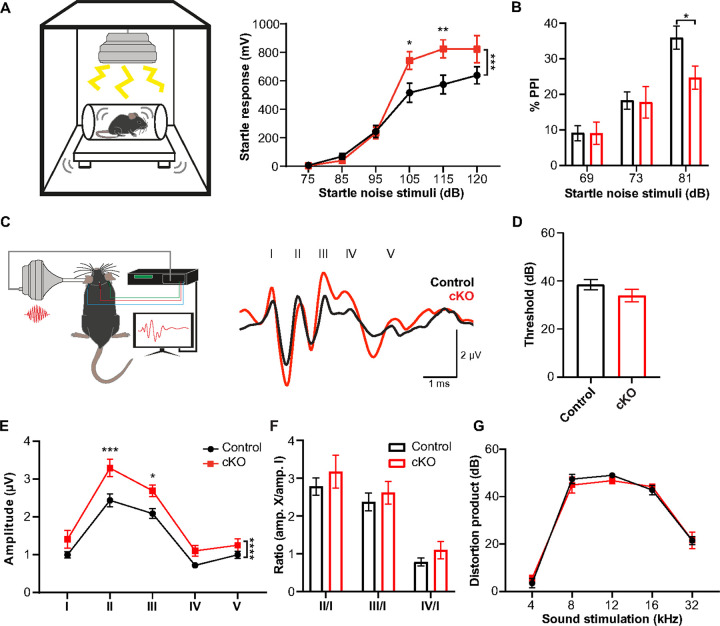
*Scn2a* cKO mice exhibit auditory hypersensitivity. **A.** Illustration of mouse acoustic startle reflex (ASR) and summary of ASR maximum startle amplitudes (mV) to sound stimuli (from 75 dB to 120 dB SPL, background = 65 dB SPL) in control (n=15) and *Scn2a* cKO mice (n=10). **B.** Pre-pulse inhibition (PPI, %) at different strength of pre-pulse stimuli (69, 73, 81 dB SPL). **C.** Illustration of mouse ABR test and representative traces of ABRs in response to click (80 dB SPL) from control and *Scn2a* cKO mice at P25. 5 distinct waves of neuronal activity corresponding to auditory nerve (I), cochlear nucleus (II), superior olivary complex(III), inferior colliculus (IV), and medial geniculate nucleus (V) in the auditory brainstem circuitry. **D.** Summary of ABR thresholds of control (n=46) and *Scn2a* cKO mice (n=29) in response to click stimuli (from 30 dB to 90 dB SPL). There was no significant difference (*p* = 0.1842, unpaired *t*-test). **E.** ABR amplitudes (μV) of wave I to V responding to 80 dB SPL clicks. The amplitude of peak II (*p* = 0.0002) and peak III (p = 0.0191) were significantly larger in *Scn2a* cKO mice (n=26) compared to control (n=39, multiple comparison from two-way ANOVA with Bonferroni correction). **F.** The ratio for the amplitude of wave I over the amplitude of each wave (II, III, and IV), evaluating central gain changes. **G.** Summary of distortion products (dB SPL) at 80 dB at various pure tone stimuli (4, 8, 12, 16, and 32 kHz). All values plotted are means per mouse ± s.e.m., * *p* < 0.05, ** *p* < 0.01, *** *p* < 0.001.

*SCN2A* is identified as a high-risk gene associated with ASD, wherein abnormal sensory processing including auditory hypersensitivity is frequently observed ^9, 28^. In our study, mice with *Scn2a* haploinsufficiency (*Scn2a*^*+/−*^ mice)^24, 29^ exhibited a distinct and pronounced startle response to sudden, loud auditory stimuli (**Supplementary Fig. 1A**). This heightened startle response is consistent with the hypersensitivity commonly observed in individuals with ASD and in ASD mouse models^24, 30^. Intriguingly, we also observed myelin deficits within the auditory nervous system of these *Scn2a*^*+/−*^ mice (**Supplementary Fig. 1B-C**). To investigate the cell-autonomous role of oligodendroglia *Scn2a* in the interplay between myelination and the auditory processing disorders, we utilized *Scn2a* conditional knockout (cKO) mice (*Pdgfra*^CreERT^; *Scn2a*^fl/fl^)^27^. First, we examined whether oligodendroglia specific deletion of *Scn2a* causes auditory processing abnormalities. In an acoustic startle reflex (ASR) test, *Scn2a* cKO mice displayed larger startle reflexes in response to stimuli above 95 dB SPL, comparing to control (n = 15 control vs n = 10 cKO mice, two-way ANOVA test, *p* = 0.0012, Fig. 1A). *Scn2a* cKO mice showed stronger startle at loud sound stimulation of 105 dB SPL (*p* = 0.0214) and 115 dB SPL (*p* = 0.0077, multiple comparison from two-way ANOVA with Bonferroni correction). The increased amplitude of the startle reflex in *Scn2a* cKO mice indicates heightened sensory perception, which is considered hypersensitivity. In addition, *Scn2a* cKO mice exhibited alterations in the pre-pulse inhibition (PPI) test. Typically, a weaker sensory stimulus (or pre-pulse) inhibits the reaction to a subsequent strong sensory event. In control, the inhibition rate drastically increased with the strength of pre-pulse stimulation. In contrast, *Scn2a* cKO mice demonstrated a diminished capacity to inhibit the startle reflex, particularly in response to the pre-pulse at 81 dB SPL sound stimulation (35.9 ± 3.22% PPI in n = 18 control vs 24.7 ± 3.19% PPI in n = 11 cKO mice, *p* = 0.0377, multiple comparison from two-way ANOVA with Bonferroni correction, Fig. 1B). The alteration in startle reflex to sound stimulation suggests that *Scn2a* cKO mice have an impaired sensory gating mechanism, a feature often seen in ASD ^13, 24^.

To ascertain the extent of auditory processing abnormalities in the sub-cortical regions of *Scn2a* cKO mice, we utilized *in vivo* auditory brainstem responses (ABRs) to assess the sum of evoked potential responding sound stimuli along the auditory pathway, particularly in the brainstem (Fig. 1C). There was no significant difference in ABR threshold (Fig. 1D) and the peak latencies between control and *Scn2a* cKO mice (data not shown). However, the amplitude of ABRs in response to 80 dB SPL (click) was significantly increased in *Scn2a* cKO mice compared to control (*p* < 0.0001, two-way ANOVA). The amplitude of peak II (2.44 ± 0.166 μV, n = 39 control vs 3.30 ± 0.231 μV, n = 26 cKO, *p* = 0.0002, multiple comparison from two-way ANOVA with Bonferroni correction) and peak III (2.09 ± 0.129 μV in control vs 2.70 ± 0.149 μV in cKO, *p* = 0.0191) were significantly larger in *Scn2a* cKO mice compared to those of control. There was a trend toward an increase, but no significant changes were observed in peak I (*p* = 0.352), peak IV (*p* = 0.3248) and peak V (*p* > 0.9999, Fig. 1E). The increased amplitude of ABRs suggested that *Scn2a* cKO mice exhibit the hypersensitivity to sound. To evaluate whether there were alterations in central gain, we calculated the ratio between peak amplitudes II to IV relative to peak I. Notably, our analysis indicated a prevalent trend where the ratios of peak II to peak I and peak III to peak I were larger in *Scn2a* cKO mice, despite the absence of statistical significance (*p* > 0.05, two-way ANOVA, Fig. 1F). We examined the possibility if the hypersensitivity could have originated from alterations in peripheral hearing sensitivity. There was no significant change in ABR threshold, peak I amplitude, and distortion product otoacoustic emissions (DPOAE, Fig. 1G), demonstrating intact peripheral hearing. Collectively, the results demonstrated robust behavioral phenotypes in *Scn2a* cKO mice, specifically auditory hypersensitivity and impaired sensory gating, mirroring sensory processing abnormalities in ASD.

### Myelin related genes were significantly downregulated in mature OL from Scn2a cKO mice

**Figure 2:**
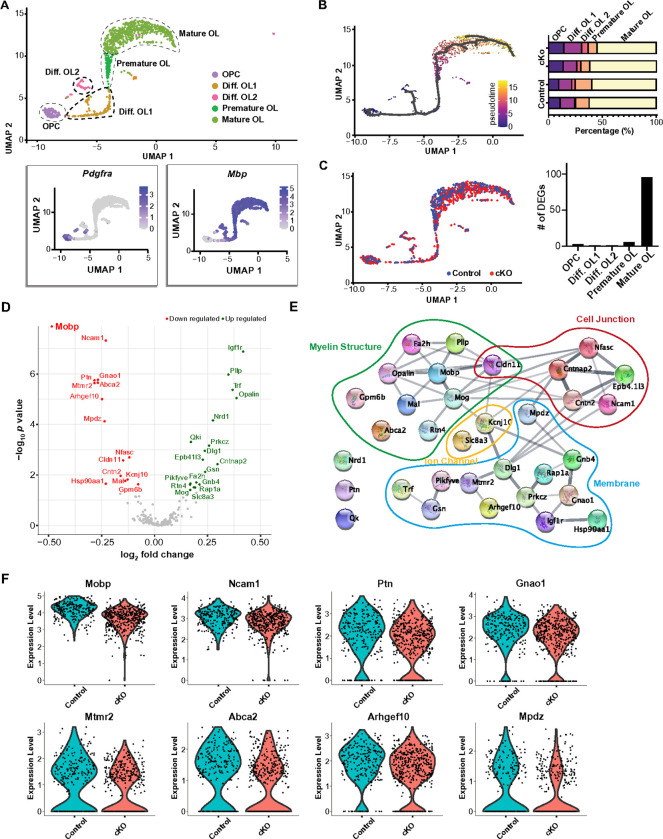
Single cell transcriptional profiles of mouse brainstems. **A.** (Top) UMAP plots of snRNA-seq dataset with clusters identified as OL lineage cells, indicated by broken-line circles; OPC, Differentiating OL1 (Diff. OL1), Differentiating OL2 (Diff. OL2), Premature OL, and Mature OL. (Bottom) Expression pattern of OL lineage marker genes such as *Pdgfra* and *Mbp*. Graded color indicators (right) showed the scaled gene expression level. **B.** The distribution of single cells colored according to the five OL lineage states identified by trajectory analysis from OPCs to mature OLs (gray line, left), and the percentage (%) of each cluster (from OPC to Mature OL) in total OLs from individual controls (n=2) and cKO mice (n=2). **C.** UMAP plots of snRNA-seq for OL lineage cells from control (blue dots) and cKO (red dots, left). The number of DEGs for each OL lineage (right). **D.** Volcano plot of differentially expressed myelin related genes (391 genes) in control and cKO mice. **E.** 33 genes showed significant differential expression (*p* < 0.05) and interconnection between those genes was detected in Sting analysis. Those genes were related to myelin structure (green line), cell junction (red), membrane protein signaling (blue), and ion channel (yellow). **F.** Violin plots of top eight genes being downregulated in *Scn2a* cKO mice; *Mobp, Ncam1, Ptn, Gnao1, Mtmr2, Abca2, Arhgef10* and *Mpdz*.

To understand the impact of *Scn2a* deletion on OL development and myelination within the auditory brainstem circuitry, we characterized the single cell transcriptional profiles of mouse auditory brainstem using single nucleus RNA sequencing (snRNA-seq). After the exclusion of low-quality cells, we obtained a total of 3083 reliable nuclei for the analysis: 989 nuclei from the control and 2094 nuclei from *Scn2a* cKO mice. Shared Nearest Neighbor (SNN) clustering identified 16 distinct clusters on Uniform Manifold Approximation and Projection (UMAP) plot, including clusters of neurons (*Rbfox3* positive), astrocytes (*Aldh1l1* positive), microglia (*Itgam* positive), and OL lineage cells (*Pdgfra* and Mog positive, **Supplementary Fig. 2**). Specifically, OL lineage cells consisted of five clusters: Oligodendrocyte precursor cells (OPCs), Differentiating OL1 (Diff. OL1), Differentiating OL2 (Diff. OL2), Premature OL, and Mature OL. To identify the OL lineage, we analyzed OL-specific genes, such as *Pdgfra, Mbp* and *Mog*, expressed in OPCs and mature OLs, respectively (Fig. 2A, **Supplementary Fig. 2**). Given the dynamic nature of OL differentiation, we utilized trajectory analysis to examine this continuous differentiation. The result of trajectory analysis from those populations displayed a narrow differentiation path connecting from OPCs to mature OLs, that similar to patterns in a previously reported study^25^. The result indicates that OL development follows a clearly defined trajectory, which was outlined by genetic markers specific to the OL lineage.

Although there was no statistically significant difference in the proportion of OL lineage cells between control and *Scn2a* cKO mice, a trend towards a reduction in the mature OL population and a slight increase in OPCs and differentiating OLs was observed in the *Scn2a* cKO mice (Fig. 2B). When OL clusters from each genotype were visualized with different colors in the UMAP scatter plot, a distinct difference was observed in mature OLs (Fig. 2C). To quantify these differences further, we identified genes exhibiting significant differential expression between control and *Scn2a* cKO mice (adjusted p with Bonferroni correction < 0.05). Notably, 96 differentially expressed genes (DEGs) were detected in mature OLs between control and *Scn2a* cKO mice (Fig. 2C, **Table S1–5**). This result reveals significant alterations in gene expression patterns within mature OLs, suggesting the cascading implications of *Scn2a* deletion in the orchestration of gene expression within mature OLs. Furthermore, to examine the expression level of myelin-associated genes in mature OLs, we employed AmiGo2, a gene ontology data base, to pick genes for comparison. Among the myelin-related genes (total 391 genes), 33 DEGs were identified (*p* < 0.05). Notably, the volcano plot showed that *Mobp*, encoding myelin associated oligodendrocyte basic protein (MOBP), was significantly decreased in *Scn2a* cKO mice (Fig. 2D). The String analysis revealed that 30 genes out of 33 DEGs have a significant interconnection and can be functionally characterized into four main categories: cell skeletal structure, cell junction, membrane, and ion channel (Fig. 2E). In addition, the most significant down regulated genes; *Mobp*^31^, *Ncam1*^32^, *Ptn*^33^, *Gnao1*^34^, *Mtmr2*^35^, *Abca2*^36^, *Arhgef10*^37^ and *Mpdz*^38^ are known to be related to myelination, cell growth, and neurological diseases (Fig. 2F). Taken together, the transcriptional profiles highlight a pronounced differential expression of myelin-associated genes in mature OLs from *Scn2a* cKO mice.

### Loss of Scn2a impairs myelination and alters axon caliber size in the auditory brainstem during postnatal development

**Figure 3:**
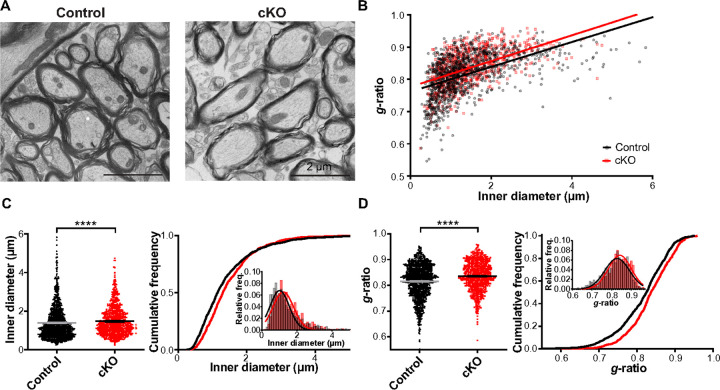
Loss of *Scn2a* in OLs increases axon caliber and decreases myelin thickness in mouse auditory brainstem. **A.** Representative TEM images of axons in the MNTB from control and *Scn2a* cKO mice at P25. **B.** Scatter plot of *g*-ratio values plotted as a function of corresponding axon inner diameter of individual axons. Datapoints from individual axons and best fit lines are shown in black for control and red for *Scn2a* cKO. The regression slopes were 0.0385 in control and 0.0399 in *Scn2a* cKO mice (*p*= 0.6295, linear regression comparison). Y-intercept was significantly increased in *Scn2a* cKO mice (0.7616 in control vs 0.7766 in cKO, *p*< 0.0001, linear regression comparison), indicating decreased myelin thickness. **C.** Summary of inner diameter of individual axons (left) and the respective cumulative frequency (right). Inset, the histogram of the distribution of inner diameter. **D.** Summary of *g*-ratio of individual axons and the respective cumulative frequency. Inset, the histogram of the distribution of *g*-ratio. Values are shown as mean ± s.e.m., **** *p* < 0.0001.

Following the gene expression analysis, we assessed the structural aspects of myelinated axons using transmission electron microscopy (TEM, Fig. 3A). This evaluation focused on determining myelin thickness and axon caliber size within axon bundles located in the medical nucleus of trapezoid body (MNTB) in the auditory brainstem. Ultrastructural analysis revealed significant myelin deficits in *Scn2a* cKO mice, as evidenced by an elevated *g*-ratio providing critical insights into the intricate alterations occurring at the structural level. The *g*-ratio is an indicator of myelin thickness measured by the inner radius (r) divided by the outer radius (R) of axon in the corresponding axon bundles. To examine alteration in distribution patter of the *g*-ratio, linear regression analysis was used in which slope and y-intercept were compared between groups. While the regression slopes of control and *Scn2a* cKO were comparable, y-intercept was significantly higher in cKO, indicating a decreased myelin thickness in *Scn2a* cKO (Fig. 3B). Axon calibers showed a larger inner diameter in *Scn2a* cKO compared to control (1.39 ± 0.03 μm, n = 1154 axons in 10 controls vs 1.48 ± 0.03 μm, n = 786 axons in 4 *Scn2a* cKO mice, *p* < 0.0001, Mann-Whitney *U*-test, Fig. 3C). The right shift of the cumulative frequency curve indicates an increase in axon caliber size in *Scn2a* cKO compared to control. Intriguingly, the *g*-ratio was significantly increased in *Scn2a* cKO, indicating a decrease in myelin thickness of axons in the MNTB area (0.82 ± 0.002 vs 0.84 ± 0.002, *p* < 0.0001, Mann-Whitney *U*-test, Fig. 3D). In addition, there was also a right shift in the cumulative frequency distribution of *g*-ratio in *Scn2a* cKO, indicating g-ratio was larger in *Scn2a* cKO across examined axons. The results suggest that the targeted deletion of Scn2a in immature OLs detrimentally affects myelination, concurrently altering the axon caliber size within the auditory brainstem. Notably, the myelin deficits identified in *Scn2a* cKO mice were consistent with significant alterations in the expression of myelin-related genes in mature OLs from snRNA-seq data (Fig. 2). For further validation, we tested effect of *Scn2a* deletion on myelination in another OL-specific *Scn2a* cKO mice, in which *Scn2a* was removed from Sox10 expressing OLs, targeting all OL lineage cells (*Sox10*^CreER^; *Scn2a*^fl/fl^)^39^, also displayed similar alterations in myelin and axon caliber size (**Supplementary Fig. 3**). The observed myelin deficits in *Scn2a* cKO mice can be attributed to both impaired OL development and alterations in myelin-associated genes. A reduction in the differentiation of OPCs to OLs, evidenced by a decreased mature OLs (CC1 + OLs) in *Scn2a* cKO mice, further supports these findings^27^. Together, these results collectively underscore the pivotal role of *Scn2a* in myelination and the maintenance of axonal integrity in the auditory brainstem.

### OL-specific loss of Scn2a alters myelinated segments and Na+ channel expression at distal axons in the MNTB

**Figure 4:**
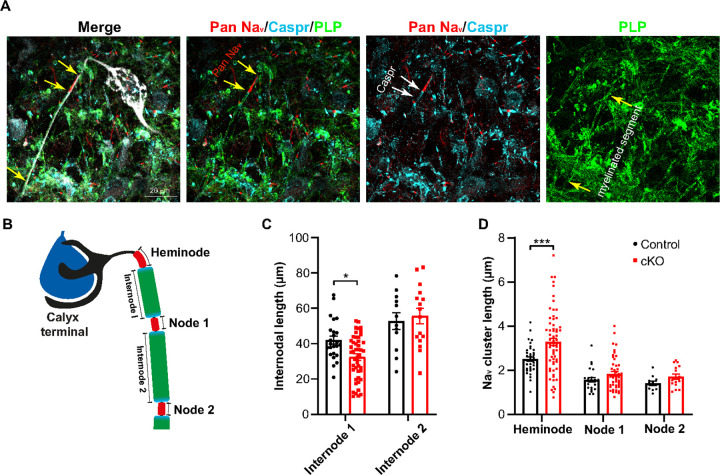
OL dysfunctions impact axonal integrity along the distal axon in the developing brainstem. **A.** The distal axons and the calyx of Held terminals from cKO mouse (at P15) were dye-filled (lucifer yellow, color-coded with white) during whole-cell recording and subsequently post-immunostaining was performed. The MNTB was immunostained with antibodies against myelin proteolipid protein (PLP, green), Pan Nav (red, yellow arrows), and Caspr (cyan, white arrows). **B.** Illustration of calyx of Held axon showing the heminode, nodes, and internodes (myelinated segments). **C.** Summary of internodal length, which was measured by distance from the heminode to node 1 (internode 1) and node 1 to node 2 (internode 2) along the distal axon from control and cKO mice (at P21). **D.** The length of Na_v_ clusters (Pan Na_v_) at heminode, node1, and node 2. Values are shown as mean ± s.e.m., * *p* < 0.05, *** *p* < 0.001.

Given the significant alterations in myelination, we postulated that myelin changes might manifest in broader structural and functional aberrations within the neural circuitry. During development, immature OLs play a pivotal role by interacting with axons, which in turn determines the length of myelinated axon segments and nodes^5^. To elucidate the consequences of *Scn2a* deletion in OLs on these interactions, we examined the long-myelinated axon extending from the cochlear nucleus to the MNTB and its terminal, the calyx of Held. Myelinated axon segments, the internode, can be visualized by myelin proteolipid protein (PLP) and caspr (a paranodal marker) flanked by two sodium channel clusters (Pan Na_v_) denoting nodes of Ranvier (Fig. 4A). Thus, we assessed the length of the internode by measuring the distance between two Na_v_ clusters (Fig. 4B). A structural analysis of the calyx axons showed a shorter internode adjacent to the heminode at the distal axon in *Scn2a* cKO mice (42.01 ± 2.31 μm, n = 24 axons from 5 control vs 32.46 ± 1.83 μm, n = 46 axons from 5 cKO mice, *p* = 0.0024, unpaired *t*-test, Fig. 4C). However, no significant difference was observed in the length of subsequent internodes (*p* = 0.6703, unpaired *t*-test, Fig. 4C). The reduced length of the last myelinated segment in *Scn2a* cKO suggests that the myelinated segments near the nerve terminal may be underdeveloped or experience delayed development in these mice^40^. Furthermore, to determine if the thinner and shorter myelin alters ion channel distribution along the distal axon in *Scn2a* cKO mice, we examined the patterns of voltage-gated sodium channel (Na_v_) expression along the calyx of Held axon. Intriguingly, we found a dispersed Nav channel expression at heminodes in *Scn2a* cKO mice (2.51 ± 0.11 μm, n = 35 axons from 5 control vs 3.29 ± 0.17 μm, n = 67 axons from 7 cKO mice, *p* = 0.0018, unpaired *t*-test, Fig. 4D). Dispersed Na_v_ channels at the heminode have also been previously observed in dysmyelinated axon terminals^5^. Nevertheless, Na_v_ clusters at other nodes did not show any significant alteration (*p* = 0.1209 for node 1 and *p* = 0.616 for node 2, unpaired *t*-test, Fig. 4D). This result highlights the pivotal role of *Scn2a* in OL development, emphasizing its influence on axon-OL interactions and Na_v_ channel distribution at the distal axons in synapse-rich areas of the auditory brainstem during postnatal development.

### The intrinsic excitability at the nerve terminal was altered in Scn2a cKO mice

**Figure 5:**
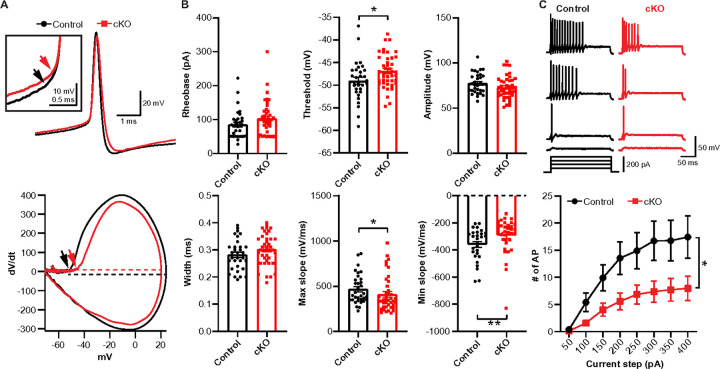
Intrinsic properties and excitability at the calyx terminal are altered in *Scn2a* cKO mice. **A.** Presynaptic APs elicited with a supra-threshold current injection (top) and their corresponding phase plots (dV/dt against membrane potential, bottom) in control (black) and *Scn2a* cKO (red). Arrows indicate the threshold of APs. AP threshold was determined by the point where dV/dt exceeds 10 mV/ms and AP amplitude was calculated from the threshold to the AP peak in the phase plot (dashed lines). **B.** Summary of AP waveform analysis; the rheobase, threshold, amplitude, width, max dV/dt, and min dV/dt. Individual datapoints are displayed in each bar graph. **C.** Top, presynaptic APs elicited by step-like depolarizing current injection. Bottom, the number of AP in response to each current injection (200 ms, from 50 pA to 400 pA), Values are shown as mean ± s.e.m., * *p* < 0.05, ** *p* < 0.01.

Alterations in ion channel distribution at the heminode influence the intrinsic properties of nerve terminal and presynaptic firing pattern. We therefore examined intrinsic excitability at the calyx terminal by recording presynaptic APs, evoked by depolarizing current injection. AP waveform analysis revealed that AP threshold was significantly higher in *Scn2a* cKO mice (−49.0 ± 0.76 mV, n = 32 cells from 17 control mice vs −46.9 ± 0.56 mV, n = 43 from 12 cKO mice, *p* = 0.0217, unpaired *t*-test, Fig. 5A, B), with no substantial changes in AP amplitude (*p* = 0.0912, unpaired t-test, Fig. 5B). The maximal dV/dt was lower in *Scn2a* cKO mice (468.1 ± 28.47 mV/ms, n = 32 control vs 411.8 ± 30.17 mV/ms, n = 43 *Scn2a* cKO, *p* = 0.0282, Mann-Whitney *U*-test), indicating a slower AP rise time in *Scn2a* cKO mice than control. Furthermore, the minimal dV/dt was significantly reduced in *Scn2a* cKO mice (−359.80 ± 22.60 mV/ms, n = 28 control vs −292.9 ± 21.75 mV/ms, n = 36 *Scn2a* ckO mice, *p* = 0.0061, Mann-Whitney *U*-test), indicating a slower repolarization. Other parameters in *Scn2a* cKO, despite not reaching statistical significance, showed trends of alteration: an increased rheobase current (*p* = 0.058, Mann-Whitney *U*-test) and a broadened AP width (*p* = 0.1437, unpaired *t*-test, Fig. 5B). An elevated threshold, a slower rising of the spike, and a slower repolarizing at the calyx terminal indicate that the nerve terminal has reduced excitability. To further support these findings, we also tested the intrinsic excitability by counting the number of APs evoked by incremental current injections. The input-output curves showed a distinct shift, demonstrating fewer APs per current step in *Scn2a* cKO (*p* = 0.0176, Two-way repeated measures ANOVA, Fig. 5C). Thus, the result suggests that alterations in Na_v_ channel expression around myelinated segments have a profound impact on presynaptic excitability in *Scn2a* cKO mice. Intriguingly, another observation is that in ~ 20% of presynaptic recordings from *Scn2a* cKO (14 of 45 cells), the calyx terminal displayed aberrant spikes or/and spontaneous spikes when the resting membrane potential was around − 65 mV (**Supplementary Fig. 4**). However, spontaneous firing and aberrant spikes have not been observed before in *ex vivo* recording from WT with the same experimental setting. The results demonstrated that myelin deficits at the distal axon caused by OL-*Scn2a* loss can generate asynchronous and abnormal spikes along the distal axon.

### Conduction failures impaired the reliability and fidelity of high frequency spikes at the nerve terminal in Scn2a cKO mice

**Figure 6:**
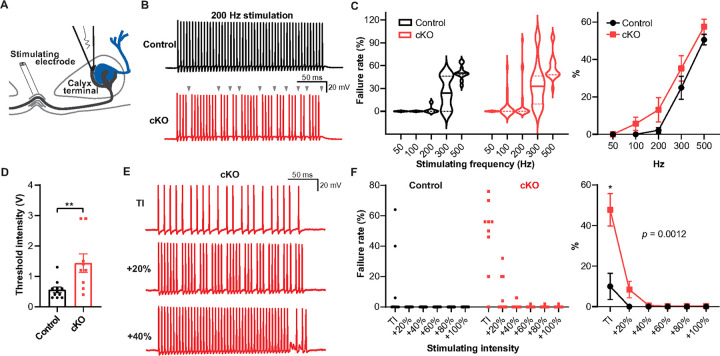
The conduction and fidelity of spikes at the nerve terminal are impaired in *Scn2a* cKO mice. **A.** Illustration of the calyx of Held recording with a bipolar stimulator placed in the midline of the brainstem. **B.** Representative traces of presynaptic AP train stimulated at 200 Hz from control (black) and Scn2acKO (red) mice. Grey arrowheads indicate AP failure during the train in *Scn2a*cKO mice. **C.** Left, violin plots of AP failure rate from control and *Scn2a*cKO mice stimulated at varied frequencies (from 50 Hz to 500 Hz). Solid and dashed lines represent median and first and third quartile, respectively. Right, average AP failure rate at varied stimulating frequencies. **D.** The average stimulating intensity to initiate a single AP (Threshold intensity, TI) from control and *Scn2a* cKO. **E.** 200 Hz AP train from *Scn2a*cKO stimulated with TI, 20% increment from TI, and 40% increment from TI. Note that increasing stimulation intensity reduced AP failures. **F.** Left, 200 Hz AP failure rate reduced by incremental stimulating intensities in control and Scn2a cKO. Circles and squares indicate individual datapoint (n = 11 cells in control vs n = 18 cells in cKO). Right, summary from the left showing average failure rate from incremental stimulating intensities (mean ± s.e.m.). * *p* < 0.05, ** *p* < 0.01.

To understand the implications of thinner myelin sheath, larger axon, and ion channel expression alterations at the distal axon observed in *Scn2a* cKO, we evaluated AP propagation along the distal axon and the fidelity of APs at the nerve terminal (Fig. 6A). In control, the calyx terminal efficiently followed high-frequency stimulation and demonstrated spikes without failures at both 50 Hz and 100 Hz. Conversely, the calyx terminal in *Scn2a* cKO mice exhibited AP failures at 100 Hz and had drastically more failures at higher frequencies (Fig. 6B, C). Among total recorded axons in response to 200 Hz stimulation, 28% of axons in the *Scn2a* cKO displayed failures compared to only 18% in control mice (data not shown). Furthermore, violin plots revealed a larger proportion of axons with over 80% failure at higher frequencies (300–500 Hz) in *Scn2a* cKO mice while no axons in control mice showed failure rate above 80% in the same frequency range (Fig. 6C). Thus, myelin defects and disruption of Na_v_ channel clustering caused by *Scn2a* loss detrimentally affect the reliability and temporal fidelity of spikes at the nerve terminal.

To discern whether AP failures at the nerve terminal are caused by conduction failures throughout the distal axon or failure to evoke AP, we tested if increasing the stimulus intensity could recover AP failures. First, we determined the minimum stimulating intensity (threshold intensity, TI) necessary to trigger a single AP for each axon. We found that threshold intensity was significantly higher in *Scn2a* cKO than in control (0.55 ± 0.09 V, n = 11 from 3 control mice vs 1.43 ± 0.30 V, n = 9 from 4 cKO mice, *p* = 0.0048, Mann-Whitney *U*-test, Fig. 6D Next, we quantified failure rate in AP train (200 Hz stimulation) with incremental intensities (Fig. 6E). The calyx terminal from *Scn2a* cKO showed significantly higher failure rates at the TI (10 ± 6.49%, n = 11 from 3 control mice vs 47.78 ± 7.95%, n = 9 from 4 cKO mice, *p* = 0.0117, Two-way repeated measures ANOVA with Sidak correction, Fig. 6F) which was recovered by increased stimulating intensity. Interestingly, all axons in control with failures at TI were able to recover by TI + 20% intensity, whereas such intensity could only recover ~ 56% of axons in *Scn2a* cKO. Most of the axons with failures in *Scn2a* cKO required at least TI + 40% to recover (*p* = 0.0012, Two-way repeated measures ANOVA, Fig. 6F). These findings were similarly observed in another OL-specific cKO mice (*Sox10*^CreER^; *Scn2a*^fl/fl^, **Supplementary Fig. 5**). Taken together with EM and immunostaining analysis, these results suggest that a shortened distal internode and thinner myelin critically impair axon conduction throughout the distal axon and cause AP failures at the nerve terminal.

### Alterations in structural and functional properties of the nerve terminal impact synaptic transmission and short-term plasticity.

**Figure 7:**
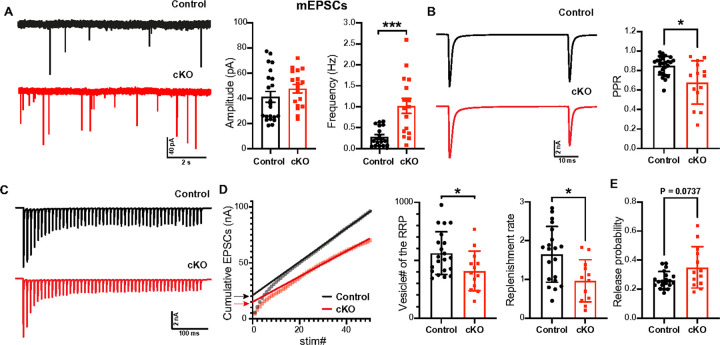
Synaptic properties in the axonal terminal were affected in *Scn2a* cKO mice. **A.** (Left) Representative trace of miniature excitatory synaptic currents (mEPSCs) from control and *Scn2a*cKO mice. (Right) Summary of mEPSC amplitude and frequency. **B.** (Left) EPSCs evoked by afferent fiber stimulation with 50 ms interval for paired pulse ratio (PPR). (Right) Summary of PPR in control and cKO. **C.** Representative traces of eEPSC trains generated at 100 Hz stimulation. **D.** (Left) Plot of cumulative EPSCs against stimulus number in control and cKO. A line fit to the steady-state points (the last 10 of 50 points) is back-extrapolated to the y-axis, and the y-intercept (arrows) divided by the mEPSC amplitude estimates the RRP size. The number of vesicles of the RRP in control and cKO (middle). The replenishment rate of vesicles, which is estimated by the slope of the linear fit (right). **E.** The release probability (Pr) is calculated by dividing the amplitude of the first eEPSC by the RRP size (the y-intercept in D). * *p*< 0.05, *** *p* < 0.0001.

Alterations in myelination and axonal properties at the nerve terminal critically impact synaptic plasticity^4, 41^. To investigate the effects of myelin plasticity changes on synaptic activities at distal axons during the critical period, we evaluated synaptic transmission and short-term plasticity at the calyx of Held-MNTB synapse using whole-cell patch-clamp recordings. In the analysis of miniature excitatory postsynaptic currents (mEPSCs), we found a significant reduction in the frequency of mEPSCs in *Scn2a* cKO mice (*p* = 0.0004 Mann-Whitney *U*-test) without changes in amplitude (*p* = 0.1989, Mann-Whitney *U*-test, Fig. 7A). The reduction in mEPSC frequency might be associated with changes in presynaptic properties. Furthermore, we recorded evoked EPSCs triggered by afferent fiber stimulation at the midline. There was no significant difference in the amplitude of a single EPSC between groups (6.56 ± 0.340 nA, n = 25 cells from 9 control, 6.45 ± 0.612 nA, n = 14 cells from 8 cKO mice, *p* = 0.9174, unpaired *t*-test, Fig. 7A). However, the paired-pulse ratio was significantly reduced in *Scn2a* cKO mice (0.852 ± 0.019, n = 25 vs 0.678 ± 0.060, n = 14, *p* = 0.0157, Mann-Whitney *U*-test, Fig. 7B), indicating an alteration in release probability (Pr). To further investigate this, we analyzed EPSC trains induced by 100 Hz fiber stimulation (50 stimuli, Fig. 7C), employing three established analysis methods for the calyx synapse^42^. Although trends were observed in Pr and RRP size, statistical significance varied (**Supplementary Fig. 6**). Across the train method analyses^43^, the vesicle number of RRPs was significantly reduced in *Scn2a* cKO mice (*p* = 0.0241, Mann-Whitney *U*-test), and Pr tended to be higher, albeit not significantly (*p* = 0.0737, Fig. 7D). Additionally, the replenishment rate was significantly lower in *Scn2a* cKO mice (*p* = 0.0107, Mann-Whitney *U*-test, Fig. 7E). These changes in the number of vesicles around the active zone, including the size of the readily releasable pool, and reduced replenishment could lead to enhanced short-term depression. Taken together, OL dysfunction led to myelin alterations at the distal axon, which in turn impacted synaptic plasticity at local synapses within the auditory brainstem circuitry.

## Discussion

Understanding the fundamental mechanisms of neuronal activity and their interplay with genetic factors provide invaluable insights for neurodevelopmental disorders, including ASD. We investigated the role of OL-*Scn2a* in the developing auditory brain from the genetic profiling to the systemic analysis. *Scn2a* cKO mice showed hypersensitivity to sound stimulation. Single-nucleus RNA sequencing revealed that the loss of OL-*Scn2a* impacts myelin-related gene expression in the auditory brainstem. Structural analysis of myelinated axons demonstrated thinner myelin and axonal alterations in *Scn2a* cKO mice. Electrophysiology and immunostaining showed that alterations in myelinated segments and nodal structure along the distal axon influenced the excitability of the nerve terminal, causing a reduced fidelity and reliability of spikes in *Scn2a* cKO mice. Additionally, those changes in the interaction between immature OLs and nerve terminal impact synaptic functions. Our findings highlight the temporal progression of myelination in early development and its potential links to the onset or severity of ASD symptoms, offering potential aids for diagnosis, prognosis, and therapeutic interventions.

Abnormal development in white matter and myelination has been found in ASD animal models and humans with autism^44–47^. However, how myelin changes are associated with compromised neural circuit function in ASD remains unclear. Disruptions to myelin can influence axonal integrity, altering ion channel distribution, and consequently affect neuronal excitability^5, 19^. Specifically, the spacing and periodicity of myelin segments determine Na_v_ channel distribution at nodes and heminodes, enabling saltatory conduction and precise neural signaling^5, 40^. In MBP deleted rats, hypomyelination led to disorganized Na_v_ channel clustering and shorter internode, which are associated with a delayed AP onset, a longer AP half width, and AP failures at the nerve terminal^5, 48^. In the cortical gray matter neuronal circuitry, cuprizone-induced demyelination caused hyperexcitability in pyramidal neurons by altering the axon initial segment (AIS) position and reducing the efficacy of AP generation^19^. The current study expands the dimensions of what have been previously reported by examining the effects of OL-*Scn2a* loss on myelin and axonal integrity specifically at distal axons near nerve terminals. We demonstrate that OL-*Scn2a* loss leads to alterations in ion channel redistribution and aberrant excitability at nerve terminals such as conduction failure and spontaneous spiking. This is particularly pertinent as *Scn2a*-expressing immature OLs are abundant in synapse-rich regions like the MNTB. Interestingly, we observed that OL-*Scn2a* loss had more pronounced effects at nerve terminals than on nodes, which differs from prior models like MBP-deleted or cuprizone-induced demyelination. In these models, mature OLs were either unable to myelinate properly or underwent cell death^5, 19^. In contrast, *Scn2a* cKO mice may have impaired interactions between immature OLs and distal axons, significantly affecting nerve terminal function including neurotransmitter release and short-term synaptic plasticity during postnatal development. Therefore, our study underscores the region-specific relationship between myelin integrity and ion channel distribution in the developing brain. We emphasize that any disturbances in myelin structure can trigger cascading effects on neuronal excitability and synaptic function in the CNS, especially at nerve terminals in the auditory nervous system.

While OL dysfunctions are highly associated with the pathophysiological process of ASD, few studies have focused on ion channels of OLs. Ion channels, including voltage-gated calcium channels (Ca_v_), Na_v_, K_v_, and inward-rectifier potassium channels (K_ir_), are expressed in OLs^49–51^. While the functions of Ca_v_ and K_ir_ channels in OLs are relatively well-defined, the roles of Na_v_ remain unclear. Our snRNA-seq data demonstrated a decline in the mature OL population coupled with an increase in OPCs and differentiating OLs in *Scn2a* cKO mice. In addition, OL-*Scn2a* deletion was found to down-regulate myelin-related genes in mature OLs. How are Na_v_1.2 channels, encoded by *Scn2a*, involved in OL maturation and myelination? One possible explanation is that the activation of Na_v_1.2 may be pivotal for triggering Ca_v_ channel activation, leading to a Ca^2+^ flux within OLs, which is involved in OL proliferation, migration, and differentiation^50^. Specifically, Ca^2+^ signaling facilitated by R-type Ca_v_ in myelin sheaths at paranodal regions, might influence the growth of myelin sheaths^49, 52^. To activate high-voltage activated calcium channels such as L- and R-Type efficiently, the activation of Na_v_1.2 channels should be required for depolarizing OL membrane to around − 30 mV ^53^. Consequently, the synergic interplay between Na_v_1.2 and Ca_v_ channels could amplify calcium signaling in OLs, initiating the differentiation and maturation processes^50^. Another possibility is that Na_v_1.2-mediated spiking in immature OLs could facilitate the release of neurotrophic factors such as BDNF, which impacts myelination via autocrine signaling. OLs are significant providers of BDNF and express the BDNF receptor TrkB^54^. Therefore, Na_v_1.2-mediated excitability of immature OLs may enhance myelination through BDNF-TrkB autocrine signaling in response to neuronal activity.

One prevalent characteristic of ASD is sensory processing disorders, notably auditory hypersensitivity. For example, *Fmrp1* KO mice showed hypersensitivity in hearing perception attributed to aberrant activity in the auditory cortex ^55, 56^. Similarly, *Shank3* KO mice displayed auditory hypersensitivity, as evidenced by amplified ABR responses and a heightened startle reflex in the ASR^57^. Here, *Scn2a* cKO mice displayed augmented ABR amplitudes and a stronger startle reflex, despite no peripheral changes. Notably, these mutant mice showed myelin defects in the CNS ^58, 59^. This raises the question: How are alterations in myelin and neuronal properties associated with auditory processing disorder? Defects in myelination within auditory pathways can lead to synchronization issues among neurons responsible for sound processing. Increased asynchronized signals can compromise the precise timing required for sound localization and the discernment of complex auditory patterns. In Scn2a cKO mice, a loss of temporal fidelity at presynaptic terminals and inconsistent spike conduction along myelinated axons was observed. Notably, ~ 20% of presynaptic recordings exhibited aberrant and asynchronous spikes at the nerve terminal in Scn2a cKO. This atypical firing of auditory neurons can contribute to auditory hypersensitivity. Neuronal firing rates were abnormally increased following demyelination, resulting in hyperexcitability of the neural circuitry^19, 60^. This hyperexcitability may be caused by alterations in sodium channel distribution and a rise in extracellular potassium along the myelinated axon^61^. Another possibility is that hypersensitivity may also emerge from changes in the intricate balance between excitatory and inhibitory regulation within the auditory brainstem. A decline in inhibitory neurons or disruptions in inhibitory inputs can induce hypersensitivity^62, 63^. In *Shank3* KO mice, hypersensitivity might be caused by diminished inhibitory regulation in the auditory circuit^57^. In addition, our snRNA-seq data suggested a downward trend in genes associated with neuronal migration in interneuron populations from *Scn2a* cKO mice (data not shown). Interestingly, the MNTB serves as a major inhibitory source, releasing GABA and glycine to various nuclei within the superior olivary complex. Consequently, abnormalities in temporal fidelity and altered neurotransmission in the MNTB may disrupt the excitatory and inhibitory balance in this subcortical circuitry, potentially leading to auditory hypersensitivity.

In conclusion, the integrity of the myelin sheath plays a pivotal role in regulating neuronal excitability and ensuring proper auditory function. Defects in myelination can create a spectrum of auditory dysfunctions, including hypersensitivity. Our results demonstrated how OL-*Scn2a* is involved in the relationship between myelin defects, neuronal excitability, and auditory pathology in ASD, potentially paving the way for targeted therapeutic interventions.

## Materials and Methods

### Animals

All procedures were approved in advance by the Institutional Animal Care and Use Committee of University of Michigan. Conventional *Scn2a* mutant mice (*Scn2a*^+/+^ and *Scn2a*^+/−^ mice)^29^ and conditional *Scn2a* knockout mice^27^ were used. To create OL-specific Scn2a knockout mice, we crossed a *Scn2a*^fl/fl^ mice with two OL specific Cre-recombinase expressing mouse lines, *Pdgfra*^CreERT^ mice and *Sox10*^CreERT^, generating double transgenic mice (*Pdgfra*^CreERT^; *Scn2a*^fl/fl^ and *Sox10*^CreERT^; *Scn2a*^fl/fl^) as described in a previous study^27^. Littermates without a Cre-recombinase but with *Scn2a*^fl/fl^ were used as control. 70 mg/kg of tamoxifen was administered via i.p. injection at postnatal days (P) 4, 6, and 8. Both male and female mice aged P15-P21 were used for immunohistochemistry, EM, and *ex vivo* electrophysiology. Mice of both sexes aged P21-P27 were used for snRNA-seq, ABR, ASR, and DPOAE.

### Slice preparation

After rapid decapitation of the mice, which were deeply anesthetized by isoflurane inhalation, the brainstem was quickly removed from the skull and immersed in ice-cold low-calcium artificial CSF (aCSF) containing the following (in mM): 125 NaCl, 2.5 KCl, 3 MgCl_2_, 0.1 CaCl_2_, 25 glucose, 25 NaHCO_3_, 1.25 NaH_2_PO_4_, 0.4 ascorbic acid, 3 myoinositol, and 2 Na-pyruvate, pH 7.3–7.4 when bubbled with carbogen (95% O_2_/5% CO_2_), and 310–320 mOsm/L. The brainstem was sectioned (200 μm thick for electrophysiological recordings and immunostaining) and the slices were transferred to an incubation chamber containing normal aCSF bubbled with carbogen, where they were maintained for 30 min at 34–35°C and thereafter at room temperature (24°C). Normal aCSF was the same as low calcium aCSF, but with 1 mM MgCl_2_ and 2 mM CaCl_2_.

### Single nuclei RNA sequencing (snRNA-seq)

A snRNA-seq was conducted using brainstem tissues from control (*Scn2a*^fl/fl^) and Scn2a cKO mice (*Pdgfra*^CreERT^; *Scn2a*^fl/fl^, P22). The brainstem was dissected and mechanically homogenized using dounce homogenizer in homogenizing buffer (250 mM sucrose, 25 mM KCl, 5 mM MgCl_2_, 10 mM Tris, 1 uM DTT and 0.1% Triton-X100) supplemented with enzymatic RNase Inhibitor (400 U/ml). 700 μl of homogenization solution was added. Homogenization involved 5 strokes of a loose pestle and 10–15 strokes of a tight one. The resultant solution was completed to 1 ml and passed through a 40μm strainer. Post centrifugation, nuclei were rinsed with PBS containing RNAse inhibitor. A subsequent filtration through a 20 μm strainer was done before resuspending in 1 ml of PBS fortified with RNAse inhibitor. For fixation, 3 ml of 1.33% PFA was added to the nuclei for 10 minutes, followed by permeabilization using 160 μL of 5% Triton X-100 for 3 minutes. After washing out the PFA, nuclei were suspended in PBS with RNAse inhibitor and quantified manually via a hemocytometer. The fixed nuclei were barcoded and prepared for library using Evercode WT Mini kit (Parse Biosciences) following manufacturer’s guidelines. The final library underwent sequencing on Novaseq 500 at Novegen (Sacramento, CA).

The raw reads were mapped and quantified using *split-pipe* v1.0.3 (Parse Biosciences). The count data was analyzed using *Seurat* ^64^ in R. To minimize artificial error, the cells with more than 1% mitochondria DNA and 5000 transcripts were considered dead cells and doublet cell, and those were excluded from further analysis. All nuclei were clustered using shared nearest neighbor (SNN) and plotted using Seurat in R. Each cluster was manually identified by examining expression of cell markers (*Pdgfra*/*Cspg4* for OPC, *Mog*/*Mbp*/*Plp1* for mature OL, *Aldh1l1* for astrocyte, *Itgam*/*Csf1r* for microglia, *Pecam1* for vascular cells, *Rbfox3* for neuronal population). To examine oligodendrocyte differentiation, the oligodendrocyte lineage clusters were isolated from the data set and trajectory analysis was conducted using *Monocle3* in R ^65^. The population of each differentiation stage of oligodendrocytes was calculated from [number of nuclei from each cluster/total number of nuclei from all of OL lineage]. To examine gene expression pattern in each cluster, the differentially expressed genes between control and cKO were obtained with *DESeq2* ^66^ method with Bonferroni multiple comparison correction in *Seurat*. For further analysis, we isolated matured OL cluster, where showed significant gene expression difference between groups, and examined expression level of myelin related genes. To list myelin related genes, Amigo2 ^67^ was used. 391 genes were searched on Amigo2 using the keyword, “myelin”. The expression level of the genes was compared between groups using *DESeq2* methods without multiple correction. The DEGs from the analysis were used gene network analysis using String ^68^. The network was manually categorized based on their function. All of data visualization was done using *ggplot2* ^69^and String.

### Electrophysiology

Slices were perfused with normal aCSF at 2 ml/min and visualized using an infrared differential interference contrast microscope (AxoExaminer, Zeiss, Oberkochen Germany) with a 63× water-immersion objective and a CMOS camera (ORCA-Flash2.8, Hamamatsu, Japan). Whole-cell patch-clamp recordings were performed in normal aCSF at room temperature (24°C) using an EPC-10 amplifier controlled by PATCHMASTER software (HEKA, Elektronik, Lambrecht/Pfalz, Germany). For recordings of eEPSCs, the pipettes were filled with a solution containing the following (in mM):130 Cs-methanesulfonate, 10 CsCl, 5 Na_2_-phosphocreatine, 10 HEPES, 5 EGTA, 10 TEA-Cl, 4 Mg-ATP, and 0.3 GTP, pH adjusted to 7.3 with CsOH. To this solution, we added 4 mM QX-314 bromide to block the voltage-activated Na^+^ channel current. Extracellular aCSF solution contained 10 mM bicuculline and 2 mM strychnine to block GABA and glycine receptors, respectively. The holding potential was − 70 mV in the voltage-clamp mode. Patch electrodes had resistances of 4–5 MW. Series resistance was < 20 MW, with 80% compensation. Afferent fibers of the calyx of Held synapses were stimulated with a bipolar electrode. For presynaptic recordings, the pipette solution contained (in mM): 125 K-gluconate, 20 KCl, 5 Na_2_-phosphocreatine, 10 HEPES, 4 Mg-ATP, 0.2 EGTA, and 0.3 GTP, pH adjusted to 7.3 with KOH. Recordings were not corrected for the predicted liquid junction potential of 11 mV. Patch electrodes had resistances of 4–5 MΩ. Current-clamp recordings were continued only if the initial uncompensated series resistance was < 20 MΩ ^4, 5^. Lucifer Yellow (1 mM, Invitrogen) was added to the pipette solution to visualize the calyx of Held terminal. Presynaptic action potentials (APs) from the calyx of Held terminal were evoked by stimulation with a bipolar platinum-iridium electrode (Frederick Haer, Bowdoinham, ME) placed near the midline spanning the afferent fiber tract of the MNTB. An Iso-Flex stimulator driven by a Master 10 pulse generator (A.M.P.I., Jerusalem, Israel) delivered 100-μs pulses at 1.2 times threshold (< 15 V constant voltage). Signals were filtered at 2.9 kHz and acquired at a sampling rate of 10–50 μs. AP waveform parameters were analyzed from the first AP induced by minimum current injection (rheobase current) and the subsequent AP phase plot, where membrane potential slop (dV/dt) is plotted against the membrane potential^70^. In both and *Scn2a* cKO mice, all cells displayed a single inflection in the rising phase of the AP, indicating APs were generated at the heminode adjacent to the presynaptic terminal. Presynaptic AP trains were obtained by averaging three sweeps (five for a single AP) in each experiment. Data were analyzed offline and presented using Igor Pro (Wavemetrics, Lake Oswego, OR).

### Auditory brainstem responses (ABRs)

To record ABR, the mice (P21-P27) were anesthetized with 3.5% isoflurane and maintained with 2.3% isoflurane during recording (1 l/min O_2_ flow rate). ABR recordings were performed in a sound attenuation chamber (Med Associates, Albans, VT). Subdermal needle electrodes (Rochester Electro-Medical, Lutz, FL) were placed on the top of the head, ipsilateral mastoid, and contralateral mastoid as the active, reference, and ground electrode, respectively. The signal differences in the ABRs between the vertex and the mastoid electrodes were amplified and filtered (100–5000 Hz). Acoustic stimuli were generated by an Auditory Evoked Potentials Workstation (Tucker-Davis Technologies [TDT], Alachua, FL). Closed field click stimuli were presented to the left ear. The signals consisted of a series of amplitude-modulated square waves (0.1 ms duration, 16/s) through TDT Multi-Field Magnetic Speakers. The sound stimuli were delivered through a 10-cm plastic tube (Tygon; 3.2-mm outer diameter) at a repeat rate of 16/s. Sound intensities ranged from 90 to 20 dB, with 5-dB decrements, and responses to 512 sweeps were averaged.

### Distortion Product Otoacoustic Emissions (DPOAEs)

Mice were anesthetized with 3.5% isoflurane and maintained with 2.3% isoflurane during recording. DPOAE recordings were performed in a sound attenuation chamber (Med Associates, Albans, VT). Acoustic stimuli were generated by an Auditory Evoked Potentials Workstation (TDT, Alachua, FLO). The ER-10B + recording microphone (Etymotic Research, Elk Grove Village, IL) with ear-tip was inserted into the ear canal. The sound stimuli were delivered through two TDT Multi-Field Magnetic Speakers connected to the recording microphone by 10-cm coupling tubes (Tygon; 3.2 mm outer diameter). Pure tones were presented at 20% frequency separation between f1 and f2 at 8, 12, 16, and 32 kHz. Sound intensities ranged from 80 to 20 dB, with 10-dB decrements, and responses to 512 sweeps were averaged. Distortion products were calculated as 2f1-f2 minus the noise floor that were detected by the recording microphone and amplified by RZ6 processor (TDT).

### Acoustic startle responses (ASRs)

Mice of either sex between ages P25-P27 were put in a Plexiglas holding cylinder located in sound-attenuated chamber using the SR-LAB startle response system (San Diego Instruments, San Diego, CA). Weights of mice were recorded to account for sex or size differences. Sound levels from the chamber speakers were calibrated with a digital sound level meter (Part Number MS-M80A, Mengshen). Each trial consisted of an initial acclimation period of 5 minutes of background level noise (at 65 dB) followed by 5 rounds of randomized noise stimuli playing for 40 milliseconds with 15 seconds of background between each noise stimuli at 65 dB (background), 95 dB, 105 dB, 115 dB, and 120 dB. Stimuli was produced by a digital signal processing-controlled system amplified and emitted by a loudspeaker. Startle responses were measured inside the sound-attenuated chamber by a movement-sensitive piezo-accelerometer platform. The maximum was used as the startle amplitude at each tone with millivolts (mV) as the unit of measurement and averaged per mouse. For each strength of stimulus, startle amplitudes were averaged across 10 trials. Startle responses to the three initial stimuli were excluded from statistical analyses.

### Pre-pulse inhibition (PPI) of acoustic startle response

PPI of ASR was examined 2 days after the ASR assessment. The apparatus and basic experimental conditions were identical to that described above. Test sessions started with the acclimatization period which included three startling stimuli (120 dB) to accustom the mice to the experimental procedure. The initial stimuli were followed by 100 trials (10 × 10 trials) presented in a random order. The PPI session involved: 10 trials with a sham stimulus (65 dB, 40 ms), three types (3 × 10) of pre-pulse trials (PP) which included only 20 ms PP stimuli (69, 73, and 81 dB), 10 pulse trials (P) which included only a pulse (startling) stimulus (120 dB, 40 ms), three types (3 × 10) of pre-pulse-and-pulse trials (PP-P) which included a 20 ms PP (69, 73, and 81 dB) followed 100 ms later by a 120-dB P stimulus. Startle responses were measured for 100 ms after the onset of the last stimulus within each trial. For each type of trial, startle amplitudes were averaged across 10 trials. The magnitude of PPI was calculated as a percent inhibition of the startle amplitude in the P trial (treated as 100%) according to the formula: [startle amplitude in P trials – startle amplitude in PP-P trials)/startle amplitude in P trials] × 100%. Startle responses to the three initial stimuli were excluded from statistical analyses.

### Immunohistochemistry

Mouse brainstem slices (200 μm) stained with tetramethylrhodamine dextran (Invitrogen) were incubated in normal aCSF bubbled with carbogen at 37°C for 30 min. All slices were fixed with 4% (w/v) paraformaldehyde in PBS for 10 min. Free-floating sections were blocked in 3% goat serum and 0.3% Triton X-100 in PBS for 30 min and incubated with the primary antibody overnight at room temperature. The following primary antibodies were used: anti-PanNa (mouse IgG1; 1:400; Sigma, Cat. #S8809), anti-Caspr (guinea pig IgG; 1:200; gifted from Dr. Manzoor Bhat, UTHSCSA), anti-PLP1 (mouse IgG2a, 1:500; invitrogen, Cat. #MA1–80034). Antibody labeling was visualized by incubation of appropriate Alexa fluor–conjugated secondary antibodies (1:500; Invitrogen) for 2 h at room temperature. Stained slices were viewed with laser lines at 488 nm, 568 nm, and 647 nm using a 40x/1.40 or 63×/1.40 oil-immersion objective on a confocal laser-scanning microscope (LSM-710; Zeiss). Stack images were acquired at a digital size of 1024 × 1024 pixels with optical section separation (z-interval) of 0.5 μm and were later crop to the relevant part of the field without changing the resolution. The confocal image stacks were analyzed using ImageJ software.

### Transmission electron microscopy

The brain was removed and immersed in ice-cold low-calcium aCSF (mentioned previously in slice preparation) and the MNTB from the brainstem was dissected at 200 μm-thick section using a microtome (VT1200s, Leica), then fixed with fixative consisting of 4% formaldehyde and 1% glutaraldehyde and stored at 4°C. Further processing was performed by the UTHSCSA Electron Microscopy Lab as previously described ^41^. Axon bundle images were analyzed at a final magnification of 8000X, with the longest length of the axon measured as the inner diameter and the inner radius divided by the outer radius as the g-ratio. Three images of axon bundles per mouse were analyzed and averaged.

### Statistical analysis

Immunostaining data were based on analyses from at least six cells in six slices from three to eleven animals. Experimental data were analyzed and presented using Igor Pro and Prism (GraphPad Software, San Diego, CA). For statistical significance, we tested the normality of the data distribution with the Kolmogorov-Smirnov test with the Dallal-Wilkinson-Lillie *p*-values using Prism 5. If a dataset passed the normality test, we used the parametric test (unpaired *t*-test for two group comparison, ANOVA for multiple comparison); for all other datasets we used the non-parametric test (Mann-Whiney U test for two group comparison). For linear regression analysis slope and intercept values were compared. Data collected as raw values are shown as mean ± S.E.M. Details of statistical methods are reported in the text. For all analyses, *p*-values < 0.05 were considered significant.

## Data Availability

Raw genetic data can be accessed on GEO NCBI with the accession number GSE252185, and analysis script can be accessible as supplementary data. All other data are available in the main text or the supplementary materials.
